# The Role of Adipose Tissue and Adipokines in Obesity-Related Inflammatory Diseases

**DOI:** 10.1155/2010/802078

**Published:** 2010-07-01

**Authors:** Carmela Rita Balistreri, Calogero Caruso, Giuseppina Candore

**Affiliations:** Immunosenescence Group, Department of Pathobiology and Medical and Forensic Biotechnologies, University of Palermo, Corso Tukory 211, 90134, Palermo, Italy

## Abstract

Obesity is an energy-rich condition associated with overnutrition, which impairs systemic metabolic homeostasis and elicits stress. It also activates an inflammatory process in metabolically active sites, such as white adipose tissue, liver, and immune cells. As consequence, increased circulating levels of proinflammatory cytokines, hormone-like molecules, and other inflammatory markers are induced. This determines a chronic active inflammatory condition, associated with the development of the obesity-related inflammatory diseases. This paper describes the role of adipose tissue and the biological effects of many adipokines in these diseases.

## 1. Introduction

Obesity may be considered as the result of a positive energy balance in conditions of energy excess. Correlated with economic, social, and lifestyle changes, it represents a common condition of different populations living in environments characterised by abundant calorie-rich food and low physical activity [1]. Hence, obesity is rapidly arriving at epidemic proportions in many parts of world and is becoming one of the major public health problems. More than 1 billion individuals are overweight and more than 300 million worldwide subjects can be classified as obese (with body mass index (BMI) of 30 kg/m^2^ or higher) [1]. More than two thirds of American population is overweight, a common condition of other Western populations [2]. In particular, the highest frequency of obesity is observed in the United States, Europe and the Middle East and the lowest in sub-Saharan Africa and East Asia [3]. In these parts of world, this condition is very alarming, because it occurs in children and adolescents [4, 5]. Hence, the current obesity may be only considered the “tip of the iceberg”, which will see young subjects develop the typical age-related diseases, because obesity predisposes to a variety of age-related inflammatory diseases, including insulin resistance (IR), type 2 diabetes, atherosclerosis and its complications, fatty liver diseases, osteoarthritis, rheumatoid arthritis, and cancer [6]. The obesity state is, indeed, characterized by what has been called “low-grade systemic inflammation”, induced by different inflammatory mediators, as demonstrated for the first time by Hotamisligil et al. in 1993 [7]. 

The growing evidence on the obesity and the associated pathologies has led to understand the role of adipose tissue as an active potential participant in controlling the physiological and pathological processes. To date, the adipose tissue is considered as an endocrine organ able to mediate biological effects on metabolism and inflammation, contributing to the maintenance of energy homeostasis and, probably, pathogenesis of obesity-related metabolic and inflammatory complications [8]. 

This review describes the role of adipose tissue and evidences the biological effects and clinical significance of many adipokines in obesity-related inflammatory diseases. 

### 1.1. Adipose Tissue: Heterogeneity and Functions

Adipose tissue is vital for the life of mammals. It represents the major source of fatty acids (FFA) in the postprandial fasting state for energy use and heat production [9]. Two types of adipose tissue are present in mammals: white adipose tissue (WAT) and brown adipose tissue (BAT) [9]. They have not only different functions, but also a different cellular composition and localization [9]. 

WAT constitutes the major component of body's adipose tissue, provides most of the total body fat and is the source of FFA, used as energy substrates for the generation through oxidative phosphorylation of adenosine triphosphate (ATP) high-energy bonds [9]. WAT is dispersed in different anatomic body's sites. Its major depots are intraabdominal around the omentum, intestines and peri-renal areas, and subcutaneous in the buttocks, thighs and abdomen [9]. Hence, it is possible to identify several local WAT subgroups, including visceral, muscle, epicardial, perivascular and kidney. Furthermore, WAT seems to have two key functions, as recently demonstrated by Worzniak et al. and Juge-Aubry et al. [8, 10]. It is involved in the control of the metabolism through energy homeostasis, adipocyte differentiation, and insulin sensitivity. Besides, it affects inflammation, through a control mechanism mediated by antiinflammatory molecules and the activation of anti-inflammatory metabolic and immune pathways [8, 10]. In addition, each local WAT subgroups have specialised roles. 

Its excessive accumulation in these body's sites might arise and determine the development of obesity and the obesity-related diseases. Much common is the WAT excess in the upper parts of body, the so-called “android obesity” or “central obesity”, which represents a strong risk factor for some inflammatory pathologies [11]. The WAT excess in other lower body's sites gives rise to the so-called “gynoid obesity” with no metabolic complications [9, 11]. 

To understand the different WAT distribution and its different link with metabolic and inflammatory complications, several theories have been advanced. Among these, two major theories, not mutually exclusive, have been considered. The first is based on the anatomy of central obesity and its capacity to drain FFA and inflammatory mediators into the portal circulation, where they can act preferentially on the liver to affect metabolism [9]. The second considers cell biology and different properties of WAT cells linked with a major or minor risk to develop metabolic and inflammatory diseases [12]. Significant differences in expression of several genes between the different body's deposits of WAT in both rodents and human have been detected [13–15]. Interestingly, a different mediator profile has also been observed between visceral and peripheral WAT. This should seem to clarify the link between central obesity and metabolic complications. Besides, it also evidences the heterogeneity in nature and kind of WAT cells [9]. Several types of cells constitute WAT: mature adipocytes and a variety of other cells (i.e., preadipocytes, fibroblasts, endothelial cells, and macrophages), usually grouped and described as the “stroma vascular fraction” [9, 11, 16]. The adipocytes, preadipocytes, and macrophages have metabolic and inflammatory functions, which render WAT able to release several mediators with different biological effects in the WAT itself or other tissues, acting in paracrine or endocrine way [8, 10, 16–20]. In particular, the macrophages are responsible for the circulating levels of specific inflammatory molecules, determining the “low-grade” chronic obesity-related inflammation [8, 11, 16, 17, 21]. 

Unlike WAT, BAT provides energy expenditure from nonoxidative phosphorylation in form of heat largely for cold adaptation [22]. The uncoupling of phosphorylation in BAT is due to the activity of uncoupling protein-1, expressed on the internal mitochondrial membrane, which by creating a proton leak exhausts the electrochemical gradient needed for oxidative phosphorylation. As consequence, BAT affects energy use by producing heat from uncoupled oxidative phosphorylation [22]. Unlike WAT, BAT also presents a smaller number of fat cells, which have richer vascular supplies with more abundant mitochondrial chromogens, responsible for the brown colour [22]. BAT with a richer vascular supply responds more rapidly to sympathetic nervous system (SNS) stimulation, which then elicits heat production, rather than ATP production, from nonshivering cold adaptive thermogenesis [22]. Hence, BAT shows a different function respect to WAT, and, in most mammals, it is responsible of the heat for fever, the arousal state from hibernation and cold-induced-thermogenesis [22]. In humans, BAT is difficult to find postnatally [22]. However, the positron emission tomography has clearly shown in adult humans metabolically active BAT depots in cervical, supraclavicular, axillary and paraventral body's regions. These depots can be induced in response to cold and SNS activation [22–24]. This highlights BAT as a potential relevant target for both pharmacological and gene expression manipulation to combat human obesity [23, 24].

### 1.2. Fat Remodelling and Regulation of Energy Homeostasis

To understand the potential mechanisms involved in energy-rich condition under overnutrition, it is necessary to known the processes of energy homeostasis. Adequate body fat and energy homeostasis are ensured through a dynamic process of fat remodelling, without excessive weight gain or loss [22]. Conditions of increased appetite and food intake determine a positive energy balance with weight gain [22]. In contrast, satiety limits food consumption determining a negative energy balance and weight loss [22]. This process is mediated through hypothalamic neuropeptide regulation of appetite and satiety [22]. Energy expenditure is regulated by central and autonomic nervous systems, which achieve a balanced energy homeostasis depending on physiological needs [25]. Hence, conditions of more energy expenditure determine WAT lipolysis and the consequent augment of FFA, or, conversely, less energy expenditure states allow an increased fat storage [22]. This is controlled by parasympathetic nervous system (PNS) and SNS. PNS facilitates the fat storage and decreases the peripheral energy use [26]. SNS stimulates lipolysis, by increasing the release of FFA for increased energy expenditure [27]. Both WAT and BAT have SNS innervations and *β*3 adrenergic receptors. BAT responds to cold-induced SNS activity with increased production of heat from uncoupled oxidative phosphorylation [27, 28]. WAT stimulated by upregulated SNS activity in response to cold increases the thermogenesis from oxidative phosphorylation of FFA within liver, muscle and fat cells, which is increased in obesity [27]. 

Thus, energy homeostasis is achieved by balancing food intake with energy use. Conditions of increased calorie intake associated with decreased energy use induce obesity. In obese conditions, BAT mass and function are strongly decreased [22]. Obese individuals who are heat intolerant are less able to dissipate heat from both WAT and BAT [22].

## 2. Obesity and Inflammation: Causes, Mechanisms and Consequences

A regulated interaction between metabolic and immunity system exists [29]. Both over and undernutrition conditions influence metabolism and immune functions [30, 31]. Malnutrition conditions can suppress immune system and increase susceptibility to infections [30, 31]. Obesity, an energy-rich condition associated with overnutrition, impairs systemic metabolic homeostasis and elicits stress [29]. Stress has been especially linked to development of visceral obesity [29]. An inflammatory process is simultaneously activated by increased WAT mass in metabolically active sites, such as WAT itself, liver and immune cells [11, 16–18, 20]. This response determines a strong increase in circulating levels of proinflammatory cytokines, hormone-like molecules and other inflammatory markers, collectively defined “adipokines” [8, 18, 19]. To counteract the obesity-related stress, hypothalamic-pituitary-adrenal axis and central and peripheral components of autonomic nervous system are activated [32]. Under stress conditions they induce physiological responses. Chronic obesity-related stress induces a prolongation of these adaptive responses. This leads to an increased level of glucocorticoid, a steroid hormone able also to induce the development and differentiation of preadipocytes, favouring consequently the further increase of WAT mass [33]. On the other hand, the secretion of pro-inflammatory cytokines by WAT may act as additional chronic stimulus for activation of hypothalamic-pituitary-adrenal axis. Hence, a vicious cycle between metabolic and immune responses in obesity state is promoted, inducing a chronic active inflammatory condition able to determine the onset of obesity-related pathologies [29, 31]. 

The causes and mechanisms involved in obesity-induced inflammatory state are not fully understood, even if the link between inflammation and obesity has been indicated by epidemiological studies from 1950s onwards. The discovery of the production of proinflammatory cytokines in the WAT, such as tumour necrosis factor (TNF)-*α*, and TNF-*α* capacity to regulate insulin action has systemically also represented a driving force in this field [7]. 

Today, current opinion proposes that, under normal WAT conditions, adipocytes store lipids and regulate metabolic homeostasis, and resident tissue macrophages, with polarization essentially of M2 type, release anti-inflammatory cytokines [21]. M2 macrophages produce arginase (enzyme involved in the inhibition of nitric oxide synthase, iNOS) and IL-10, IL-1Ra anti-inflammatory cytokines [21, 34, 35]. In contrast, M1 type macrophages have a specific surface marker (CD11c+) and release iNOS and classical proinflammatory cytokines [21, 34, 35]. Normal WAT is, so, characterised by an anti-inflammatory tissue milieu able to protect from the development of obesity-related inflammation and IR, most likely also due to activity of members of peroxisome proliferator-activated receptor-(PPAR)s (particularly PPAR-*α* and -*γ*) and liver X receptor-(LXR) families, molecules involved in nutrient transport and metabolism and able to antagonize inflammatory activity [36, 37]. To contribute to this physiological WAT condition is a new adipokine, the lipocalin-2 (LCN2). LCN2 upregulates PPAR*γ*, increases the release of adiponectin and also antagonizes TNF-*α* effects on inflammatory and metabolic gene expression in adipocytes and macrophages. Conversely, knocking down LCN2 expression, using lentiviral shRNA gene silencing, results in decreased expression of PPAR*γ* and its target genes, adiponectin, and leptin. Hence, LCN2, seems to act as an antagonist to the effect of inflammatory molecules on inflammation and secretion of adipokines [38].

In obesity conditions, WAT becomes inflamed, state determined by a crosstalk principally between adipocytes and macrophages [8, 16, 21]. The obesity-related inflammatory state occurs in several sequential stages, characterised by a cellular WAT composition remodelling. An increase in number (hyperplasia) and size (hypertrophy) of adipocytes, a macrophage infiltration, and fibrosis characterise WAT in obesity human conditions [39–45]. Adipocyte hypertrophy is induced by two factors: increased fat storage in fully differentiated adipocytes and increased expression of proinflammatory mediators [43–45]. On the other hand, hypertrophic adipocytes shift the immune balance towards the production of proinflammatory molecules [40, 46–49]. The shift in the cytokines profile creates a tissue milieu responsible of the strong modification of the WAT macrophages pool from activated M2 type to classically-activated M1 type [21, 34, 35, 40, 46–49]. In addition, the M1 macrophage WAT pool considerably increases because of the differentiation of monocytes recruited in inflamed WAT. In obese WAT, macrophages also aggregate in “crown-like structures” constituted by necrotic-like adipocytes and adipocyte cellular fragments [46–51]. An increased infiltration of macrophages, indeed, occurs in the inflamed WAT, preferentially into visceral WAT, which contributes of the WAT inflammation state and its exacerbation [10, 16, 17, 20, 21, 34, 35, 46, 47]. Several chemokines, chemokine receptors, and adhesion molecules are involved in this process [50]. It seems also to be directly correlated with both hyperplasia and hypertrophy of adipocytes and inflammatory mediator production [10, 16, 17, 20, 21]. However, the mechanisms responsible for attracting monocyte/macrophage cells and their entry into the fat mass remain unclear. The group of Sengenès et al. has recently evidenced a key role of endothelial cells in the control of the inflammatory WAT process. It has also been described the potential involvement of WAT-endothelial cells as further factors involved in the regulation of macrophage phenotype in the “inflamed fat tissue” [48]. Inflammatory WAT cytokine profile seems to be responsible of the activation of endothelial cells and their expression of a series of adhesion molecules involved in the recruitment of monocyte/macrophages. Furthermore, it has been demonstrated an association between angiogenesis and adipogenesis [52]. 

Concerning the shift of cytokines (M2/M1 cytokine profile), the exact mechanisms involved have not yet been clarified [21, 34, 35, 40, 46–49]. It has been proposed that adipocytes from different body depots show differences in their inflammatory phenotype, with visceral fat characterised by more inflammatory phenotype respect to subcutaneous fat, as discussed above [9, 12]. Besides, the increased obesity-associated preadipocyte differentiation process seems also to contribute to this inflammatory cytokine profile [9, 12]. However, human WAT macrophage subsets show no strict M1 or M2 polarization, as recently demonstrated by Bourlier et al. [40] and Zeyda et al. [21]. 

Furthermore, another characteristic phenomenon, the local hypoxia induced by hypoperfusion due to rapid fat mass expanding seems to contribute to WAT inflammatory state [53–55]. Adipose hypoxia, indeed, induces the release of proinflammatory mediators [53–55].

However, on the whole, these observations give no a clear dissection of the cause and effect relationship between obesity and inflammation. On the other hand, it has, recently, been demonstrated that increased adipose tissue mass is not essentially related with WAT inflammation state, as observed in two studies performed in adiponectin transgenic mice and lipocalin-2 knockout mice [56–58]. This suggests that, although obesity is directly linked to inflammation, the specific role of adipokines and related pathways might be clarified.

## 3. Molecules Produced by WAT: “Adipokines”

WAT releases hundreds of biologically active molecules, the “adipokines”, including more than 50 cytokines, chemokines, hormone-like factors and other mediators [8, 18, 19] Not exclusively produced by WAT cells, these mediators are released by other different body's tissues and organs with functions unrelated to those within WAT [18, 19]. Adipokines affect appetite and satiety, glucose and lipid metabolism, blood pressure regulation, inflammation and immune functions [8, 18, 19]. Precisely, they work as a network to regulate inflammation, insulin action, and glucose metabolism locally and systemically. This adipokine/cytokine networking system is altered in obesity, contributing to inflammation state and impaired adipocyte metabolism. However, how adipokines and cytokines coordinately regulate obesity-related inflammation and metabolism is not clearly understood [8, 18, 19]. 

Different mechanisms are involved in the adipokine secretion. The production of inflammatory adipokines (such as proinflammatory cytokines, chemokines, molecules associated with thrombosis, and hypertension, etc.) seems to be complex and involves several inflammatory pathways, activated by both extracellular mediators and intracellular stressors. Among extracellular factors, the FFA are the primary inductors of these pathways [59]. In human obesity, they are chronically elevated (by determining lipotoxicity), because of blunted incapacity of insulin to inhibit the lipolysis and the excessive consumption of dietary lipids [60]. 

Innate immunity receptors, such as Toll-like receptor (TLR)-4 and -2, are expressed in WAT (particularly by adipocytes, preadipocytes, macrophages, and endothelial cells) and are involved in this obesity-related inflammatory process. Their expression is increased and induced in obese subjects [60, 61]. FFA and other molecules produced by hypoxic conditions during obesity activate these receptors, particularly TLR4 [60, 61]. Lipopolysaccharide (LPS) is another factor able to activate TLR4 [62]. A key source of LPS is the gut microbiota [59]. It is continually produced within the gut by death of Gram-negative bacteria and is absorbed into intestinal capillaries to be transported by lipoproteins [63, 64]. On the other hand, it has been observed that a high-fat diet given to mice increases the proportion of gut LPS [63, 64]. These data indicate the gut microbiotia may have an important role in the induction of chronic obesity-related inflammation [63].

Hence, FFA, particularly via TLR4 induce the pro-inflammatory adipokine production in adipocytes [65, 66]. FFA also activate macrophages, referentially of the CD11c+ subset, through the TLR4 pathway, exacerbating their pro-inflammatory activity [66–68]. Furthermore, a paracrine loop between hypertrophied adipocytes and macrophages has been evidenced, able to induce a vicious circle of inflammatory exacerbated WAT state [68]. This paracrine loop involves FFA and TNF-*α*. As a result, macrophages secrete the pro-inflammatory TNF-*α*. TNF-*α* in turn, acting particularly on TNF-*α* receptor 1 subtype, induces inflammatory changes in hypertrophied adipocytes as well as increased release of FFA [68]. In addition, this paracrine cross-talk could be further improved in obese subjects through the adipocyte hyperresponsiveness to TNF-*α* and subsequent hyperactivation of inflammatory pathway [52]. FFA may also be active on the adipocyte in an autocrine way to evoke an inflammatory state and chemokine/adipokine overproduction at least in part via TLR4 [69]. This autocrine mechanism has been proposed to be an initial event of the inflammatory WAT cascade, but this issue is still controversial.

In obese WAT the cells and the intracellular organelles are also exposed to increased stress, mainly as a result of metabolic overload [31]. In particular, mitochondria and the endoplasmic reticulum appear to be the most sensitive organelles to metabolic stressors [70]. In addition, the development of hypoxic conditions in the expanded WAT during obesity results in an increased production of reactive oxygen species and the corresponding development of oxidative stress [70].

Signals mediated by both extracellular and intracellular factors culminate predominantly in the activation, principally via TLR4 receptor, of NF-*κ*B transcriptional factor, responsible of the production of inflammatory mediators, as well as the direct inhibition of insulin signaling [60, 61, 71]. Hence, NF-*κ*B pathway represents the crucial and major factor responsible of obesity-induced inflammation. To amplify inflammation-mediated inhibition of insulin action are also other pathways, such as those mediated through the suppressor of cytokine signaling-(SOCS)-proteins and iNOS [72, 73]. 

In contrast, anti-inflammatory adipokine molecules seem to be released through the activation of different transcription factors induced via PPAR-*γ* and LXR receptors, as mentioned above [36–38]. In conditions of overnutrition, fatty acid-binding proteins, FABPs, likely sequester ligands of PPAR-*α* and LXRs and induce no activation of these transcription factors [74]. 

In this paper, the attention has particularly been focussed on adipokines involved in the obesity-related inflammatory diseases. The biological effects of metabolic and inflammatory adipokines are reported in Tables 1 and 2.

## 4. Role of Visceral Obesity in Ageing and Obesity-Related Inflammatory Diseases

**Figure 1:**
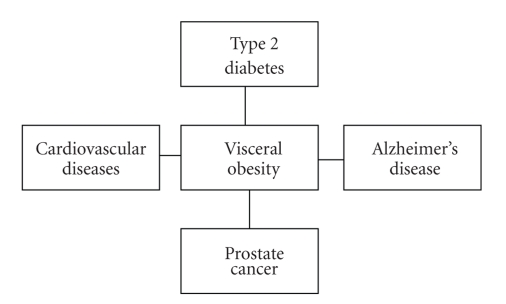
A common yet preventable risk factor for these multifactorial age-related diseases is the visceral obesity. Metabolic syndrome is also associated with obesity. It assembles some abnormalities, including insulin resistance, hyperinsulinemia, hypertension, and dysplipidemia, all risk factors directly associated with both type 2 diabetes and cardiovascular diseases.

Several pathologies are associated with obesity, such as type 2 diabetes and cardiovascular diseases (CVD) [6]. More recently, the obesity-related risk has also been extended to cancer, including prostate, breast, liver, kidney, colon, ovarian and endometrial cancers [75–81]. The obesity-related diseases are characterised by inflammatory pathophysiology induced by several risk factors (environmental stressors, genetic factors, etc.) and an onset generally correlated with ageing process. Epidemiological studies have revealed that a common yet preventable risk factor for these diseases is the increase of the visceral fat, a characterised hallmark of ageing in humans (Figure 1) [8, 11, 12, 82]. Using either waist circumference and/or waist-to-hip ratio as a proxy of visceral obesity, the role of visceral fat as stronger risk factor for these diseases, than BMI or other fat depots, has been confirmed.

 Obesity-related risk is not only limited to these diseases, but also to cognitive decline, Alzheimer's disease (AD) and disability, as recently demonstrated [6, 83–85].

Recent evidence also reports an association of obesity with increased risk of disease specific and all-cause mortality, and with a reduced life expectancy [82]. For example, the group of Fontaine reported that Caucasian men and women with a BMI >40 and age range 20–29 years, could expect a reduction in remaining years of life expected by approximately 6 and 12 years, respectively [86]. An increment of mortality and a reduction of life expectancy correlated with obesity, especially in old subjects, has been, indeed, proposed. On the other hand, obesity, and precisely visceral obesity, seems to accelerate ageing process. It has been demonstrated that obese women have telomeres of 240 bp shorter lean women of a similar age [87]. In view of the hypothesis that telomere length in vivo represents cellular turnover and exposure to oxidative and inflammatory damage, this difference in telomere length between being lean and being obese might correspond to 8.8 years of ageing [87]. 

The increased evidence on visceral obesity, as a stronger predictor of these diseases, has led to assess the mortality risk correlated with abdominal obesity [88–90]. In 2008, a large European study reported that both general (BMI) and abdominal adiposity (waist circumference; waist-to-hip ratio) are strong predictors of mortality risk [91]. However, the importance of visceral obesity was most remarkable among persons with a low BMI [91]. 

The question of visceral fat, as factor capable of reducing life expectancy, has been recently clarified in an animal model study [92]. The extrapolation of its data in humans suggests the key role of visceral fat and the possibility through its depletion to favour the survival and, hence, longevity, as demonstrated in calorie restriction rats [92]. In human beings, its beneficial effects might be greater, since humans visceral fat depots have direct portal access and, so, a greater potential to harm the liver [82, 92]. This has recently led Huffman and Barzilai to hypothesise the presumed link of visceral fat with both age-related diseases and lifespan. Accumulation of visceral fat represents a greater risk for the development of IR and other processes of metabolic syndrome than other fat depots due to its anatomical location, high lipolytic rate and secretion of inflammatory adipokines. This determines some specific perturbations to tissue including hepatic IR, impaired glucose uptake by skeletal muscle and increased basal lipolytic rate and WAT free fatty acid release. The long-term consequences are an increased risk for obesity-related inflammatory diseases and mortality and a reduced lifespan [82]. 

### 4.1. Evolutionary Speculations about the Link between Obesity and Obesity-Related Diseases

The association of obesity with obesity-related inflammatory diseases might be explained through evolutionary speculations. It is appropriate, hence, to consider the fundamental biological necessities for the survival of an organism: (1) the ability to resist to starvation and (2) to evoke an efficient immune response to pathogens. To this aim, several metabolic and immune pathways and nutrient- and pathogen-sensing systems have been selected and evolutionarily conserved throughout species. The metabolic systems have been selected to assure energy efficiency through the storage of excess calories particularly under intermittent food uptake conditions. In contrast, under continuous overnutritional conditions, these systems induce a fat excess, as no advantageous state associated with the onset of several chronic disorders, such as obesity and its complications. At the same time, the necessity to defend against the infections has determined the selection of strong immune components, particularly induced by the epidemic and pandemic infections [93, 94]. The combination of these efficient systems has likely created a fundamental biological instrument able to store energy and to evoke immune-inflammatory responses. Its existence is showed by the several pathways with metabolic and immune functions evolved from common and ancestral units. A characteristic ancestral unit is the body fat of *Drosophila*, having metabolic functions and an intimate control of immune responses. It has given rise after about 600 million years to homologous mammalian tissues, such as haematopoietic and immune systems, liver and adipose tissue [95–98]. These mammalian tissues have conserved their development heritage and show metabolic and immune cellular components with an architectural organization able to have immediate access into blood vessels. Hence, they have continuous and dynamic interactions with other important metabolic and immune sites, such as pancreas. This contributes to understand the mechanisms involved in metabolic diseases, such as type 2 diabetes [99]. They have common regulatory molecular pathways and pathogen-sensing systems able to regulate both metabolic and immune functions. Characteristic example is the TLR4-NF-*κ* *β* pathway, evolved by Drosophila homologous Toll and able to mediate efficient immune responses also metabolically or nutritionally induced and lipid-related pathways able to respond to the energy necessities of particular conditions, such as during the induction of immune or inflammatory response [66, 93, 95]. 

These observations suggest a fine balance between metabolic and immune systems. Its dysfunction is dangerous and responsible of the development of some diseases. In particular, overnutritional conditions, fruit of current nutritional habits and lifestyles of most modern Western populations, are responsible of the development of the obesity-related inflammatory diseases [100]. 

In the light of these observations and suggestions, we described the role of adipokines in the pathophysiology of obesity-related inflammatory diseases.

## 5. Adipokines and Obesity-Related Inflammatory Diseases

### 5.1. Metabolic Syndrome and Cardiovascular Diseases

The term “Metabolic syndrome” (MS) assembles some abnormalities, including visceral obesity, dyslipidemia, hyperglycaemia and hypertension. The criteria to define MS have been established by International Diabetes Federation (IDF) [97]. In the IDF consensus, MS is defined by the presence of visceral obesity plus two of the described components [101, 102]. The presence of any two of the four next components is also required: elevated circulating levels of triglycerides, reduced levels of HDL-cholesterol, high blood pressure and impaired fasting glycaemia [101, 102]. The IDF eventually recommends other criteria to diagnose MS, such as increased levels of circulating inflammatory and/or thrombotic markers (CRP, SAA, TNF-*α*, IL-6, and PAI) or reduced levels of anti-inflammatory molecules, such as adiponectin. This syndrome is currently considered one of the major public health challenges, as demonstrated by two large studies performed, respectively, in 2.600 American individuals (age range 25–64 years) and 3000 European subjects (age range *∼*55 years) [103]. In the two populations, the 25%–40% and 30% (of an Italian cohort), respectively, were affected by MS [103]. 

The MS pathophysiology is complex and different adipokines seem to be involved. Several reports have demonstrated in MS patients increased IL-6 levels related to BMI [100, 101]. In particular, the involvement of IL-6 in IR and its complications has been evidenced, even if its role remains controversial [104–106]. The mechanisms involved, indeed, are not fully clear. However, IL-6 seems to induce IR, impairing hepatic signaling through the increased expression of SOCS-3 and affecting the phosphorylation of insulin receptor substrate 1 (IRS-1) and the transcription factor PKB/Akt [104–106]. SOCS-3 has the capacity to bind and to inhibit the insulin receptor and to induce the proteosomal degradation of IRS proteins. Using 3T3-L1 adipocytes, it has been demonstrated the IL-6 capacity to induce partial resistance in insulin-dependent glucose uptake through down-regulation of the phosphorylation of IRS-1 and the expression of IRS-1 and Glucose transporter 4 (GLUT–4) [10, 66–68, 107]. Furthermore, in 3T3-L1 adipocytes, IL-6 reduces the activity of lipoprotein lipase-LPL [52, 107]. 

TNF-*α* seems also involved in MS. High TNF-*α* levels have been observed in MS subjects [100]. The relationship between high levels of TNF-*α* and MS is related to the TNF-*α* capacity to induce a c-Jun NH2-terminal kinase to mediate a serine phosphorylation of IRS-1. This determines the inhibition of normal tyrosine phosphorylation of IRS-1 and downstream insulin signaling [104]. 

Furthermore, in obesity, an overexpression of angiotensinogen and an increased activity of vasoconstrictor renin-angiotensin system have been demonstrated [108]. This phenomenon seems also to contribute to the alteration of insulin sensitivity and to increase the incidence of type 2 diabetes and MS [109]. 

Recent evidence also demonstrates the association of elevated levels of systemic inflammatory molecules, such as SAA and CRP, with type 2 diabetes and MS [110]. 

It has also been demonstrated the role of leptin in the MS pathophysiology. It evokes a condition, which affects insulin sensitivity and induces IR. In particular, leptin induces in hypothalamus the release of “anorexigenic peptides” (i.e., proopiomelanocortin and corticotrophin-releasing hormone) and, reciprocally the inhibition of “orexigenic peptides” (i.e., neuropeptide Y and agouti-related protein), thereby limiting food intake [111]. In obesity conditions, hypothalamic resistance to leptin has been found and ascribed to reduced transport of leptin across the blood-brain barrier and to increased levels of SOCS-3 and ER stress, which inhibit leptin signaling [112, 113]. This evokes profound changes in energy balance and hormone production via the hypothalamus, analogous to those induced in response to fasting. Hence, a response of adaptation to low levels of leptin is induced, determining overfeeding and inhibition of energy expenditure, thyroid and reproductive hormones, and immunity. Hypothetically, this response may be evolved as a protection against the threat of starvation [111]. In obese subjects, these changes determine reduced energy expenditure and to regain weight, associated with lipid accumulation [114]. Ectopic lipid storage (in liver, epicardial, and muscle fat) is also induced in obese conditions because of leptin resistance, which may in turn further impair insulin sensitivity [52]. Like leptin effects, another adipokine, resistin, seems to mediate IR. Its role may be evidenced in rodents, as suggested by an interesting theory [18, 19, 52]. However, successive studies in both rodents and humans have reported contradictory data [18, 19]. 

An interesting role in the MS pathophysiology and its complications seems mediated through a more recent discovery adipokine, visfatin, which its plasma levels are correlated to lipid metabolism and inflammatory response [18, 115]. It mediates a nicotinamide adenine dinucleotide (NAD) biosynthetic activity in pancreatic *β* cells [116]. Hence, visfatin acts as nicotinamide phosphoribosyltransferase (Nampt), the rate-limiting enzyme that converts nicotinamide (a form of vitamin B3) to nicotinamide mononucleotide (NMN), a NAD precursor [112]. It has been reported a decline with advanced age of Nampt-mediated systemic NAD biosynthesis, able to determine a reduced sirtuin-1 activity. This mechanism might contribute to decreased function of pancreatic *β* cells in aged subjects [117]. 

Furthermore, another adipokine recently identified with a key role in insulin resistance is LCN-2, as demonstrated in LCN-2 knockout mice [57]. 

In contrast, a protective role of adiponectin against MS (and the other obesity-related pathologies) has recently been demonstrated [118]. This molecule reduces M1 macrophage functions, by inhibiting phagocyte activity and release of IL-6 and TNF-*α*, and increases the IL-10 and IL-1Ra production in adipocytes and macrophages [10, 18, 19]. Apelin also reduces the MS risk. In obesity, increased plasma and WAT levels of apelin have been detected [18, 19]. TNF-*α* seems to be the responsible of these increased levels both in plasma and WAT [18, 19]. This molecule seems to reestablish glucose tolerance and increased glucose utilization, as demonstrated in a mice study [119]. These data should suggest the use of this molecule in the treatment of IR. Studies on animal models particularly in the apelin-knockout mice have evidenced that loss of apelin determines heart diseases in response to pressure overload [120]. 

Growing evidence highlights the link of systemic-obesity inflammatory state with both CVD onset and CVD risk [110, 121]. Several studies have demonstrated a link between WAT excess and CVD mortality in young (particularly in adolescents) and old subjects [110, 121]. Furthermore, a relationship between WAT excess and coronary artery calcium (a marker of coronary atherosclerosis) measurement has been reported [122]. Imaging approaches have also been confirmed this association [122]. How obesity determines the CVD development, it is until now not clearly understood. Complex and numerous obesity-mediated mechanisms are identified, as well as the CVD risk obesity-related factors (including hypertension, IR and dyslipidemia). Systemic and WAT adipokines seem also to affect vessel wall, by determining adverse effects [110, 121, 122]. Precisely, proinflammatory cytokines, hormone-like molecules and other WAT adipokines act in the liver, causing changes in the production and the release of lipoproteins, coagulation factors and inflammatory molecules [110, 121, 122]. In particular, they induce an increase of very-low-density lipoprotein, apolipoprotein B (apoB), and triglyceride secretion [123]. These liver-released molecules act on endothelial, arterial smooth muscle and macrophages cells, by inducing atherogenic effects on the vessel wall through the regulation of their gene expression and functions [110, 121, 122]. In addition, visceral fat seems to be particularly involved in the activation of these pathways [122, 123]. 

Interestingly, among adipokines, leptin mediates different effects on cells of vessel wall. It evokes on endothelial cells oxidative stress, increased production of adhesion molecules and chemokines and proliferation [110, 121, 122]. For example, an increased blood release of MCP-1 in obese condition has been observed. It seems to increase the number of CD11c+ monocytes, favouring the binding of monocytes/macrophages to the vessel wall [124]. Acting also on smooth muscle cells, leptin induces their migration, proliferation, and hypertrophy [110, 121, 122]. It also induces a further activation and cytokine production of macrophages, neutrophils, and T cells and it seems also involved in the calcification of cells of vessel wall and the thrombosis through the increase of platelet aggregation [110, 121, 122]. These effects are also indirectly mediate of leptin through leptin resistance (mentioned above). 

Resistin induces similar effects to those of leptin. In human, levels of resistin seem to be positively associated with coronary atherosclerosis [125]. It induces on endothelial cells an increased expression of adhesion molecules, proinflammatory cytokines, and pentraxin [125].On smooth muscle cells, it evokes their migration. An increased expression of CD36 on macrophages seems to be also mediated by resistin, facilitating lipid accumulation and formation of foam cells [126, 127]. On macrophages it also mediates an increased production of proinflammatory cytokines, through via TLR4 and NF-*κ*B pathway [128]. 

Among the adipokines of recent discovery, visfatin, and apelin seem to have a key role in the CVD pathophysiology. Visfatin has a key role in plaque destabilization, associated with its increased expression in macrophages of human unstable carotid and coronary atherosclerosis [129]. In contrast, its plasma levels are negatively associated with vascular endothelial function [130]. Paradoxical data reported by van der Veer et al. have demonstrated that visfatin can, however, prolong the life of human smooth muscle cells [131]. 

Unlike visfatin, apelin is associated with a positive hemodynamic profile and has positive inotropic effects in normal and failing rat hearts and in isolated cardiomyocytes [132–134]. In patients affected by single atrial fibrillation and chronic heart failure, reduced plasma apelin levels have been found [135, 136]. In vessel wall and cardiovascular tissue of rats, apelin production seems to be upregulated by hypoxia and ischemic cardiomyopathy, likely as a compensatory mechanism [137, 138].

In contrast, adiponectin seems to induce beneficial effects. Its levels are positively correlated with HDL levels, and negatively with triglyceride levels, IR, and systemic circulating inflammatory markers [139, 140]. Furthermore, a negative correlation between adiponectin and coronary artery calcium has been observed [141]. It also promotes several anti-atherogenic and anti-inflammatory effects on vessel cells: it downregulates the expression of adhesion molecules on endothelial cells [142]; it decreases endothelial oxidative stress and increases eNOS activity [143]; in smooth muscle cells it inhibits proliferation by suppressing the release of growth factors [144]; and in macrophages it reduces lipid accumulation and the expression of scavenger receptors [145]. 

Furthermore, CRP, usually increased in CDV, has atherogenic effects on vessel wall [146, 147]. This atherogenic effect is also increased by other WAT molecules, such as AGT, angiotensin-converting enzyme and PAI-1. AGT II has vasoconstrictive actions and also promotes systemic inflammation [121, 122]; AGT contributes to the activation of renin-angiotensin system (RAS) and both these molecules induce a hypertensive response [117, 118]. PAI-1 seems to be involved in atherothrombosis [121, 122].

### 5.2. Obesity and Alzheimer Disease

AD is a heterogeneous and progressive neurodegenerative disease which in Western societies mainly accounts for clinical dementia [148]. The AD prevalence is below 1% in individuals aged 60 years, but shows an almost exponential increase with age, so that, in the Western world, in people aged 85 years or older the prevalence is between 24% and 33%. It prevalence is expected to quadruple by the year 2047 in the United Stated [149]. 

There is currently no cure for AD and its pathogenesis remains the subject of many theories involving genetic as well as environmental factors. Recent mounting evidence has been supposed the involvement of modifiable risk factors in AD neurodegeneration, such as lifestyle factors. Among these, obesity represents an AD risk factor. Several potential mechanisms seem to link obesity with AD: hyperglycemia, advanced glycosylation products, adipokine action, and the influence of obesity on vascular risk and cerebrovascular disease. 

IR and hyperinsulinemia seem to represent the key causes for the development of some age-related diseases, such as AD [6]. Recently, a role of insulin in AD neurodegeneration has been reported [150]. Precisely, insulin, crossing the blood brain barrier from periphery to central nervous system, seems to compete with A*β* amyloid peptide for insulin degrading enzyme (IDE) in the brain, including also the hippocampus [151]. In contrast, insulin produced in the brain seems to have an advantageous effect on amyloid clearance [152]. Opposing effects seem to be mediated by conditions of peripheral hyperinsulinemia. They seem to determine the inhibition of brain insulin production, which in turn results in impaired amyloid clearance and a higher AD risk [152]. These data suggest that reducing peripheral hyperinsulinemia and increasing brain insulin levels, beneficial effects might be attained on AD neurodegeneration. Therapy strategies able to reduce blood insulin levels in humans have been demonstrated to affect cognition and levels of amyloid *β* in the cerebrospinal fluid, supporting the potential direct role of insulin in AD [153, 154]. 

Hyperglycemia seems to be also responsible of the increased levels of advanced glycosylation end products (AGEs). An increased glycation of amyloid *β* has been demonstrated to improve its aggregation *in vitro*. Furthermore, AGE receptors seem also to be specific cell surface receptors for amyloid *β*, thus potentially facilitating neuronal damage [155]. 

Concerning the role of adipokines in AD, it is not clear whether their involvement in the AD pathophysiology are direct or associated with IR and hyperinsulinemia. On the other hand, systemic inflammation seems to be a risk AD factor [156]. Some studies have proposed a direct action of adipokines in AD neurodegeneration. For example, leptin seems to affect CA1 nucleus of the hippocampus [157]. Several effects of leptin on the brain development and potentially on brain health in cognition and ageing have also been observed. This evidences the capacity of leptin to affect the function of the hypothalamus and learning and memory processes controlled by the hippocampus [157]. Leptin receptors have been observed in the hippocampus, hypothalamus, amygdala, cerebellum, and brain stem, supporting its capacity to mediate regulatory mechanisms [157]. A direct interaction between leptin and adiponectin and hypothalamic nuclei has been evidenced [157]. However, other roles of leptin and related adipose-derived factors in the AD brain are not clear [158]. Fasting plasma leptin has been inversely correlated with grey matter volume in areas of the brain in which obese have reduced grey matter in comparison with lean individuals [159].

Another potential link between obesity and AD is cerebrovascular disease (CD). CD and stroke are associated with a higher AD risk [160–162]. Their direct action on amyloid cascade, however, is not clear. Current opinion proposes CD as additional brain damage to amyloid neurotoxicity [161, 162]. However, RAS system seems to link obesity with CD and AD. RAS system regulates the blood pressure. Both human brain and WAT express RAS, with WAT RAS involved in adipocyte growth, differentiation and metabolism [163, 164]. The RAS activation takes place when blood pressure is low. In this state, the formation of Angiotensin II is evoked. Its interaction with specific receptors induces the activation of RAS, determining an increased of blood pressure. In the brain, angiotensin II continues its conversion to angiotensin IV, which enhances learning and memory in animal models [165, 166].

Another potential mechanism theoretically involved in AD neurodegeneration is hypercholesterolemia. To this aim, some prospective studies have examined total LDL and HDL cholesterol levels as possible risk AD factors. Contrasting data have been reported. The association of cholesterol with dementia may vary depending upon when cholesterol is measured in the life-span and/or relative to the course of disease. High cholesterol may be a risk factor if measured in midlife many years before clinical onset, but then as the disease pathology progresses, cholesterol levels may fall such that it appears that high cholesterol is protective. Two Finnish studies have, indeed, observed that high total cholesterol levels in mid-life are associated with an increased risk of AD more than 20 years later [167, 168]. In contrast, no association has been found cross-sectionally [164]. Two studies each with more than 25 years of follow-up, did not find an association between mid-life total and HDL cholesterol and incident AD [169, 170]. Three other studies in elderly populations also did not find associations between LDL and/or HDL cholesterol and incident AD after follow-ups of 2 years and 7 years [171, 172]. In fact, one of these studies reported an inverse association between total cholesterol and AD, such that those in the lowest quartile had the greatest risk [168]. Similarly, higher cholesterol levels have been reported to be associated with a reduced risk for AD [173, 174].

### 5.3. Obesity and Prostate Cancer

Prostate cancer (PC) is the most common cancer in Western elderly male populations. Its incidence increases rapidly in men over 50 years of age [175]. The development of PC is based on the interaction between genetic factors and the host exposure to environmental factors, such as infectious agents, dietary carcinogens and hormonal imbalances. In this complex situation, chronic inflammation seems to play a key role [176–181]. 

As reported above, the risk associated with obesity has also been extended to several malignancies. Its role in PC aetiology is less clear [182]. Data on the association between obesity and PC incidence are inconsistent, and in some studies obesity is associated with an increase in risk of low-grade tumours. The reasons of these contrasting results may be due, in part, to variation of the methods of anthropometric measurement, such as BMI and the waist-to-hip ratio. However, a recent study has revealed visceral fat accumulation as specific risk factor for PC [6, 76]. More consistently, it has recently been suggested that obesity reduces the risk of nonaggressive PC disease and increases the risk of aggressive PC disease [182]. Hence, it is possible that rather than increasing the absolute risk of PC development, obesity may be associated with the progression of latent or microscopic PC to clinically significant and metastatic PC. Furthermore, the differential effects of obesity on PC subtypes suggest aetiological heterogeneity of these tumours and complex interaction between androgen metabolism and several putative risk factors, including IR, diabetes, inflammation, and genetic susceptibility, on PC risk [182].

The molecular mechanisms liking obesity and PC pathophysiology are numerous and occur at several levels. A first mechanism seems correlated to sex steroid pathways [182]. However, the relationship between sex steroid hormones and obesity is complex and biological processes involved are unclear. Current opinion suggests a decline in men of serum testosterone levels in obese conditions [183]. In addition, increased peripheral aromatization of androgens to oestrogens, correlated with fat overload, is also involved in the decline of androgens [182]. On the other hand, it is well-documented an age-related decrease of serum testosterone levels [183]. However, testosterone seems to induce differential effects. Recent data have shown that higher serum levels of total testosterone are associated with a reduced risk of high-grade PC, but with an increased risk of low-grade PC [184]. This emphasizes the complex relationship between obesity and serum sex steroid, and their differential effect on PC, but further supports the differential effect of obesity on PC subtypes.

Another mechanism is correlated to capacity of obesity to modify the production of other hormones, such as insulin-like growth factors (IGFs), having mitogenic properties. An additional mechanism is mediated by adipokines. It has been reported that adipokines may modulate the biological behaviour of PC cells. In particular, leptin, IL-6 and TNF-*α* seem able to enhance tumour growth [185]. The association between systemic leptin levels and PC has been analysed in several studies. The obtained data have reported a positive association between high leptin levels and the risk of large volume prostatic tumours [185]. Stattin et al. have evidenced an association between moderately high leptin levels and later PC development [185]. Furthermore, the association between leptin and PC seems particularly confined to male subjects having a with waist-to-hip ratio >0.87. This datum evidences the interaction of leptin with other molecules correlated with abdominal obesity, such as sex hormones bioavailability and IGF-1 levels [185]. Another study has demonstrated in a relatively small number of PC patients an association between serum leptin levels and prostate specific antigen and Gleason score [185]. In vitro studies have evidenced a role of leptin in PC carcinogenesis and its capacity to promote the proliferation of androgen-independent PC cell lines [185]. In vitro, it has been also observed the capacity of leptin to induce vascular endothelial cell proliferation, and in vivo angiogenesis, key processes involved in cancer progression, invasion, and metastasis [185]. The proliferative response of PC cells to leptin has been shown to involve intracellular signaling molecules such as phosphatidyl-inositol 3-kinase (PI3-K) and c-Jun NH2-terminal kinase (JNK) [185]. Alterations in these signaling pathways are not only critical in processes of prostate carcinogenesis and malignant transformation, but also important in obesity, diabetes, and IR [185]. 

High serum IL-6 levels are also associated with PC [185]. A role of IL-6 has been suggested in the early stages of prostate carcinogenesis [185]. 

Difference effects seem to be mediated by the other adipokines, such as adiponectin proposed as an anticancer factor in some tumours, PC included [181]. In support of this, significant lower levels of adiponectin have been observed in PC patients respect to subjects with benign prostatic hyperplasia or healthy controls [185]. This study also evidences a negative association between plasma adiponectin and Gleason score and PC stage. Adiponectin receptors have been found in both benign and malignant human prostate tissue [185]. In the LNCaP and PC3 PC cell lines, it has been evidenced that androgens, oestrogen, TNF-*α*, leptin and adiponectin seem all to act and regulate adiponectin receptors. These results might to suggest a complex role of adiponectin in the PC carcinogenesis, mediated through its interaction with sex hormones and cytokines.

## 6. Conclusions: Possible Strategies for New Therapeutic Treatments for Obesity-Related Inflammatory Diseases

Human visceral obesity represents one of the major risk factors for obesity-related diseases. Possible strategies for the prevention and the development of new therapeutic treatments are, hence, crucial medical challenge. 

In researching potential strategies, crucial questions remains open. It is not clear whether effectively obesity inflammatory state determines a metabolic deterioration. Furthermore, it is not also clear whether inflammation can simply be considered a state activated by altered nutrient clearance. 

To date, the literature evidence leads to consider the regulator molecular pathways, evolutionary well-conserved and able to control the evocation of immune responses and metabolic processes and, as possible ways for therapeutic approaches. However, their selection is difficult, as well as their manipulation with possible pharmacological agents to interfere with immune and metabolic systems, without to determine severe consequences on key mechanisms of organism. A possible candidate might be the TLR4-NF-*κ*B pathway, having the role of hub in the induction of both metabolic and inflammatory processes, as described. Hence, its antagonists might be used to block the release of metabolic and inflammatory adipokines. On the other hand, TLR4-NF-*κ*B pathway has a key role in the pathophysiology of age-related inflammatory diseases, such as CVD, AD, and PC, as we have recently demonstrated [62, 176, 186, 187]. 

Other possible pathways might be the lipid-related pathways, such as PPAR-*α* and LXR pathways. It is already established with success that the use of thiazolidinediones or statins (ligands of this pathway and insulin sensitising compounds) is able to regulate lipid metabolism and to induce anti-inflammatory effects. 

Another possible way for the development of possible pharmacological approaches for inflammatory obesity-related diseases might involve the adipokines, even if several their effects and functions remain unclear. In this case, the strategy might hypothetically have as aim the control of the bioavailability of some adipokines, such as leptin and adiponectin, in obese conditions. Exogenous administration of adiponectin might counteract the consequences of obesity state, such as leptin-induced inflammation, or activate its antiatherogenic, vasoprotective and anticancer actions. Another alternative avenue might be the inhibition of leptin receptors through monoclonal antibodies or mutant leptin. Other possible targets might be pro-inflammatory cytokines and chemokines or their receptors, through the use of their agonists or monoclonal antibodies. 

In summary, these observations emphasize the necessity to discover the metabolic and immune pathways, including also the molecules involved in metabolic and pathogen sensing systems, involved in the delicate balance of interplay between metabolic and immune systems. This might be useful to clarify and to understand the mechanisms induced and to open possible ways for therapeutic approaches able to enhance the capacity of endogenous molecules to prevent stress and inflammatory responses induced by metabolic signals.

## Figures and Tables

**Table 1:** Adipokines involved in energy balance/metabolism.

Name	Cell type expression	Biological effects
*Leptin*: no glycosylated peptide hormone of 16 kDa encoded by the obese (ob) gene, discovered in 1994 by Zhang et al. [18, 19]	*Adipocytes*: synthesis induced by food intake, eating-related hormones, energy status, and sex hormones (being inhibited by testosterone and increased by ovarian sex steroids) and several proinflammatory mediators (being increased or inhibited by proinflmmatory cytokines with acute or chronic action)	Satiety signal with direct effects on the hypothalamus; stimulates lipolysis; inhibits lipogenesis; improves insulin sensitivity; increases glucose metabolism; and stimulates fatty acid oxidation. Hence, leptin operates as adipostatin and general inductor of energy reserve, being involved in glucose metabolism, synthesis of glucocorticoids. However, it is also known its involvement in other processes, such as the proliferation of lymphocytes (particularly CD4+) and induction of Th1 response, cytokine production, phagocytosis, and regulation of hypothalamic-pituitary-adrenal-axis, reproduction, angiogenesis, and oxidative stress. Collectively, these functions consent to define leptin as a cytokine-like hormone characterised by pleiotropic propriety [18, 19]

*Adiponectin: *a protein of 30-kDa with a structural homology with collagen VIII and X and complement factor C1q. Once synthesised, it forms trimers which then oligomirize to constitute polymers composed of 4 to 6 trimers. Trimers, hexamers, and high molecular weight (HMW) 12 to-18 mers of adiponectin are present in circulation [18, 19]	*Adipocytes*	Increases fatty acid oxidation with reduction in plasma fatty acid levels; decreases plasma glucose levels; increases insulin sensitivity; anti-inflammatory, antioxidant, antiatherogenic and anticancer properties through the inhibition TNF-*α*-mediated of NF-*κ*B pathway

*Resistin**: ***a member of resistin-like molecule (RELMs) family, called “resistin” for its capacity to induce IR in mice. Resistin is also known as member of molecule ‘‘found in inflammatory zone” (FIZZ)—family characterised by four members, characterised by a conserved 11-cysteine pattern at the C terminus. Resistin or FIZZ-3 was initially discovered in mice [18, 19]	*Adipocytes and M2 macrophages *	Induces severe hepatic insulin resistance-increased rate of glucose production in rat (increased resistin plasma concentrations in diet-induced obese mice, but reduced mRNA levels in WAT of obese rodents; stimulates lipolysis); functions controversial in humans

*Adipsin: *(also called in human complement factor D46) is a rate-limiting enzyme in the alternative pathway of complement activation [18, 19]	*Adipocytes and M2 macrophages*	Stimulates triglyceride storage, inhibits lipolysis

*Apelin: *a bioactive peptide, representing endogenous ligand of orphan G-protein-coupled receptor, APJ, homolog to angiotensin II receptor [18, 19]	*Adipocytes and stromal vascular cells (in particular macrophages)*	Reduces food intake (?); inhibits glucose-induced insulin secretion; antagonize angiotensin II effects in atherosclerosis inducing NO production and inhibiting angiotensin II cellular signaling (? However, there are contrasting literature data).

*Visfatin: *An insulin mimetic adipokine recently discovered and released prevalently by visceral WAT. Visfatin is also identical to pre-B-cell colony-enhancing factor (PBEF), a cytokine that has been observed increased both in bronchoalveolar lavage fluid in animal models and in neutrophils in septic conditions Under endotoxin stimulation, PBEF/visfatin is produced by neutrophils, inhibiting neutrophil apoptosis [18, 19]	*Adipocytes* * Under * * endotoxin stimulation, PBEF/visfatin/NAMPT is also produced by neutrophils, inhibiting neutrophil apoptosis *	Insulin-mimetic effects; hypoglycaemic effects by stimulating glucose uptake; promotes insulin sensitivity; proadipogenic and lipogenic action. It also induces chemotaxis and the production of IL-1*β*, TNF-*α*, and IL6 in CD14+ monocytes and increases proliferative responses in lymphocytes. In addition, visfatin seems to have a nicotinamide adenine dinucleotide (NAD) biosynthetic activity in pancreatic *β* cells [49]. Hence, visfatin acts as nicotinamide phosphoribosyltransferase (Nampt), the rate-limiting enzyme that converts nicotinamide (a form of vitamin B3) to nicotinamide mononucleotide (NMN), and a NAD precursor [51].
*Vaspin: *a serpin (serine protease inhibitor) [18, 19]	*Adipocytes*	Improves insulin sensitivity; suppresses the production of resistin, leptin, and TNF-*α*

*Omentin: *a secretory protein, recently identified as a new adipokine It is codified by two genes (1 and 2) [18, 19]	*Stromal vascular cells (in particular macrophages)*	Enhances insulin-stimulated glucose transport in subcutaneous as well as omental adipocytes; modulation of insulin action

*Lipocalin-2 (LCN2), also known as 24p3 or neutrophil gelatinase-associated lipocalin (NGAL): *a recently indentified adipokine of the superfamily of lipocalins. It is a 25kDa secretory glycoprotein, originally identified in mouse kidney cells and human neutrophil granules. In adipose tissue, it is highly expressed in vivo and in vitro, and its secretion is regulated by the activation of inflammation and infection [58].	*Adipocytes and macrophages, but also neutrophils, hepatic and kidney cells*	Has different actions, such as apoptosis and innate immunity; affects glucose metabolism and insulin sensitivity; seems to have dual effects on inflammation: pro- and anti-inflammatory effects. So, increased levels of LCN2 in obesity and IR may constitute a protective mechanism against inflammation [58]

*Retinol binding protein-4 (RBP4): *this protein belongs a the superfamily of lipocalins. And it is a specific carrier for retinol in the blood [58].	*Adipocytes *	Promotes IR and the type 2 diabetes [58]

**Table 2:** Adipocytokines, chemokines, vascular proteins, and other proinflammatory markers produced in WAT and systemic sites and involved in the inflammatory-obesity responses.

Name	Cell type expression	Biological effects
*Proinflammatory cytokines *		

*IL-6: *one the crucial pro-inflammatory mediator, secreted by several body's cell types (monocytes, adipocytes, endothelial cells, fibroblasts, etc.) [10, 11]	*Stromal vascular fraction*: the 90% WAT IL-6 production comes from these cells. Under the obesity conditions, both adipocytes and macrophages are the principal responsible of WAT derived IL-6, although the stimuli for the induction of IL-6 production seem to be different.	Decreases insulin and leptin signaling; induces the hepatic release of acute-phase proteins, such as C-reactive protein, and the hypothalamic induction of fever; seems to have a controversial role in insulin resistance: it seems to impair hepatic signaling through the increased expression of SOCS-3 impairing the phosphorylation of insulin receptor substrate 1 (IRS-1) and the transcription factor PKB/Akt. Furthermore, down-regulates the expression of IRS-1 and Glucose transporter 4. In addition, SOCS-3 has the capacity to bind and to inhibit the insulin receptor and to induce the proteosomal degradation of IRS proteins. IL-6 also induces fatty acid oxidation and lipolysis [102]

*TNF-*α*: *another remarkable proinflammatory cytokine [10, 11]	*Adipocytes and M1 macrophages*	Induces IR and increases lipolysis in adipocytes; decreases adiponectin and increases IL-6 expression. TNF-*α* should also play an atherogenic role inducing an increased expression of adhesion molecules in vascular wall, increasing the scavenger receptor class A expression and oxidised LDL uptake in macrophages and stimulating their infiltration in vascular wall

*IL-1*: Another pro-inflammatory cytokine, member of IL-1 family (IL-1*α*, IL-1*β*, and IL-1Ra) [10, 11]	*M1 Macrophages *	Induces fever, acute-phase proteins, proliferation of fibroblasts, smooth muscle cells, and production of antibodies, cytokines, and angiogenesis, metastasis, and cartilage breakdown. It also appears to affect glucose homeostasis and insulin sensitivity through central and peripheral mechanisms. IL-1 also mediates direct effects on adipocytes, decreasing the expression and the activity of LPL, increasing lypolisis and affecting adipocyte differentiation through inhibition of PPAR receptors

*Anti-inflammatory cytokines*		

*IL-1Ra: *a cytokine antagonist able to limit inflammation, competing with IL-1 for binding to its receptor without inducing a signal [10, 11]	*M2 macrophages and hepatic cells as an acute-phase protein under systemic inflammation stimuli*	Produced in response to stress and by M2 macrophages to create an anti-inflammatory WAT milieu in physiological condition. High serum levels of IL-1Ra are associated with insulin resistance

*IL-10: *an anti-inflammatory cytokine inhibiting the production of several proinflammatory cytokines (IL-1, IL-6, and TNF-*α*), chemokines and increasing the levels of anti-inflammatory cytokine such as IL-1Ra [10, 11]	*Adipocytes and M2 macrophages*	Produced by M2 macrophages to create an anti-inflammatory WAT milieu in physiological condition. In obesity, high levels of IL-10 have been observed

*Proinflammatory chemokines *		

*IL-8: *a proinflammatory chemokine [10, 11]	*Stromal vascular cells*	Induces the migration of different cell blood types, such as monocytes, particularly in inflammatory conditions. In obesity, high IL-8 levels have been observed and increased levels of IL-8 mRNA have been detected principally in visceral WAT. They seem correlated to fat mass and BMI

*Mcp-1 (CCL2): *key chemokine involved in recruitment of monocytes/macrophages and in monocyte tissue infiltration. Its levels conspicuously increase under IL-1, TNF-*α*, and LPS stimuli, while under normal conditions are undetectable [10, 11]	*Adipocytes/M1 macrophages*	Increases lipolysis and leptin secretion; decreasesinsulin-stimulated glucose uptake; (increased plasmaconcentrations in obesity; disturbs insulin sensitivity)
*Adipokines associated with thrombosis and hypertension and other inflammatory markers *		

*PAI-1: *a serine protease inhibitor (serpin) with the physiological function to inhibit plasminogen activation [10, 11]	*Stromal vascular cells with visceral WAT secretion more elevated than subcutaneous WAT*	Inhibits plasminogen activation. Elevated PAI-1 levels determine a pathological condition characterised by hypofibrinolysis and a prothrombotic state It affects cellular matrix degradation, smooth muscle cell migration and angiogenesis, determining the development of atherosclerosis. In obese conditions, PAI-1 seems to contribute directly to obesity complications, such as atherothrombosis, insulin resistance and type 2 diabetes

*Angiotensinogen (AGT)*: the precursor of vasoactive peptide angiotensin II (Ang II), a component of vasoconstrictor renin-angiotensin system (RAS) [10, 11]	*Stromal vascular cells and adipocytes, with visceral WAT secretion more elevated than subcutaneous WA*	Linked to vascular inflammation (increased plasmalevels in obesity) and increased blood pressure

*C-reactive protein (CRP): * one of the acute-phase proteins in inflammation. It is a member of short pentraxins produced in the liver in response of IL-6 [10, 11]	*Hepatic cells, human mature adipocytes, but not preadipocytes, under inflammatory stimuli, including lipopolysaccharide (LPS), TNF-* *α* *, and resistin*	Endothelial dysfunction, adhesion molecules expression, Tissue factor production, PAI-1upregulation, mononuclear cells recruitment, adhesion, activation and cytokine production, ROS and MMPs production, uptake of oxLDL, foam cells formation, proliferation, migration, ROS production, MMPs, MCP-1, and iNOS expression

*Serum amyloid protein (SAA):* constitute a family of lipoproteins involved in the transport of cholesterol and the host defence alarm system [10, 11].	*Hepatic cells, adipocytes *	SAA are not only inflammatory markers induced by IL-6, but also represent inflammatory mediators able to induce inflammatory events in leucocytes. In particular, SAA proteins can mediate chemotaxis of monocytes into WAT with hypertrophic adipocytes and at the same time to increase the expression of adhesion molecules in endothelial WAT cells. SSA proteins seem responsible of increased incidence of cardiovascular diseases in obese individuals. They are able to interact with high-density lipoprotein (HDL)-receptor competing with HDL, and thereby inhibit the HDL-mediated clearance of cholesterol, increasing the development of atherosclerotic lesions.
